# Emergency government response team for global infectious disease outbreaks—a Japanese challenge

**DOI:** 10.1186/s41182-016-0035-4

**Published:** 2016-10-25

**Authors:** Koji Wada, Yasuo Sugiura, Hidechika Akashi, Tamotsu Nakasa

**Affiliations:** Bureau of International Health Cooperation, National Center for Global Health and Medicine, Tokyo, Japan

The Ebola virus outbreak in West Africa made it clear that international emergency assistance must be strengthened [1]. The Japanese government has contributed to combating infectious disease outbreaks by extending emergency aid to affected countries through international organizations, such as the World Health Organization (WHO) and the Red Cross, and by providing emergency relief directly to affected countries. The Japanese government also dispatches individual experts on infectious diseases to affected regions through the Global Outbreak Alert and Response Network of the WHO [2]. During the Ebola virus outbreak, Japan contributed approximately USD173 million in emergency aid and through various technical cooperation programs, and dispatched 20 experts to the affected countries through the WHO [3].

In October 2015, the Japan Disaster Relief (JDR) Infectious Diseases Response Team was established to respond to large-scale infectious disease outbreaks at the request of governments requiring technical assistance with these outbreaks [4], although a scheme for responding to natural disasters, the JDR, has been in place since 1987 [5]. The Japanese government offered to take the initiative in protecting human security during the G7 Ise-Shima Summit in 2016, and this new scheme was established in accordance with this policy [6]. The aim of this letter to the editor was to share the characteristics of the scheme and benefits which were identified based on the first dispatch to Democratic Republic of the Congo (hereinafter DRC).

The JDR Infectious Diseases Response Team is a national team that collaborates with the relevant governmental agencies in the affected countries and international partners, including the WHO. The team would offer effective assistance in those areas specified by the governments of the affected countries. During the mission, the team will also be supported by the country that requested assistance, which will provide the necessary coordination in the field.

Another strength of the team is that it is supported by multidisciplinary and comprehensive expertise. The JDR Infectious Diseases Response Team includes five areas of expertise: epidemiology, public health, clinical issues (treatment and infection control), laboratory testing and diagnosis, and logistics. Japanese experts from different organizations voluntarily register for membership of the team; approximately 200 experts had registered as of August 2016.

The government of the DRC declared a yellow fever outbreak in three provinces on June 20, 2016, after identifying the first case of yellow fever on March 22, which was imported from the Republic of Angola, where a yellow fever outbreak had been identified [7]. On May 19, the WHO declared that the yellow fever outbreak in DRC and Angola was a public health concern requiring international assistance [8]. In July 2016, the JDR Infectious Diseases Response Team was dispatched on the first mission to respond to the yellow fever outbreak [9].

Based on the request of the government of the DRC, the team comprised experts in public health, infectious diseases, laboratory testing and diagnosis, and logistics, together with an official from the Japanese Ministry of Foreign Affairs, totaling 17 members. By providing technical assistance, the team facilitated the resumption of the IgM test for yellow fever, which had been suspended because the necessary reagents had been exhausted at the DRC National Reference Laboratory. Consequently, the surveillance of yellow fever was strengthened and the number of confirmed cases was updated regularly. The team also supported the reactive vaccination campaign with technical assistance, and joined the DRC Ministry of Health in recommending that a further mass vaccination campaign be implemented to include about 10 million people in Kinshasa in the middle of August 2016. The recommendations made were (1) to implement an effective communication strategy on the mass vaccination campaign through the media and public advertisements before and during the campaign; (2) to establish a detailed logistical plan and ensure that resources were available before the campaign, to procure the necessary consumables and conduct supervisory visits to the vaccination sites during the campaign; and (3) to monitor any serious adverse events caused by vaccination, which although rare, cause a certain number of people to require medical interventions.

This new scheme would be an addition to the Japanese government’s contributions to promoting global health security. In reviewing the first mission in the DRC, we also identified ancillary benefits: (1) Japanese experts gaining experience in combating diseases not often found in Japan, such as yellow fever and (2) strengthening of friendships among infectious disease experts from the DRC and Japan, which leads to further international technical cooperation. This scheme will provide mutual benefits both for countries with serious infectious disease outbreaks and for Japan.

## Abbreviations

DRC: Democratic Republic of the Congo
JDR: Japan Disaster Relief
WHO: World Health Organization
